# Detection of Abiotic Stress in Potato and Sweet Potato Plants Using Hyperspectral Imaging and Machine Learning

**DOI:** 10.3390/plants14193049

**Published:** 2025-10-02

**Authors:** Min-Seok Park, Mohammad Akbar Faqeerzada, Sung Hyuk Jang, Hangi Kim, Hoonsoo Lee, Geonwoo Kim, Young-Son Cho, Woon-Ha Hwang, Moon S. Kim, Insuck Baek, Byoung-Kwan Cho

**Affiliations:** 1 Department of Biosystems Machinery Engineering, Chungnam National University, Daejeon 34134, Republic of Korea; qkralstjr208@naver.com (M.-S.P.); akbar.faqeerzada@gmail.com (M.A.F.); hhh342@naver.com (S.H.J.); zxcvkhk@gmail.com (H.K.); 2 Department of Biosystems Engineering, College of Agriculture, Life, and Environment Sciences, Chungbuk National University, 1 Chungdae-ro, Seowon-gu, Cheongju 28644, Republic of Korea; hslee202@chungbuk.ac.kr; 3 Department of Bio-Industrial Machinery Engineering, College of Agriculture and Life Science, Gyeongsang National University, 501 Jinju-daero, Jinju-si 52828, Republic of Korea; geonwookim@gnu.ac.kr; 4 Department of Smart-Agricultural Industry, School of Agricultural Life Sciences, Gyeongsang National University, 501 Jinju-daero, Jinju-si 52828, Republic of Korea; protaetiacho@gnu.ac.kr; 5 Division of Crop Physiology and Production, National Institute of Crop Science, Rural Development Administration, 181 Hyeoksin-ro, Iseo-myeon, Wanju-gun 55365, Republic of Korea; hwangwh@korea.kr; 6 Environmental Microbial and Food Safety Laboratory, Agricultural Research Service, United States Department of Agriculture, Beltsville, MD 20705, USA; moon.kim@ars.usda.gov (M.S.K.); insuck.baek@usda.gov (I.B.); 7 Department of Smart Agricultural Systems, Chungnam National University, Daejeon 34134, Republic of Korea

**Keywords:** hyperspectral imaging, abiotic stress detection, machine learning, band selection, crop health monitoring

## Abstract

As climate extremes increasingly threaten global food security, precision tools for early detection of crop stress have become vital, particularly for root crops such as potato (*Solanum tuberosum* L.) and sweet potato (*Ipomoea batatas* L. Lam.), which are especially susceptible to environmental stressors throughout their life cycles. In this study, plants were monitored from the initial onset of seasonal stressors, including spring drought, heat, and episodes of excessive rainfall, through to harvest, capturing the full range of physiological and biochemical responses under seasonal, simulated conditions in greenhouses. The spectral data were obtained from regions of interest (ROIs) of each cultivar’s leaves, with over 3000 data points extracted per cultivar; these data were subsequently used for model development. A comprehensive classification framework was established by employing machine learning models, Support Vector Machine (SVM), Linear Discriminant Analysis (LDA), and Partial Least Squares-Discriminant Analysis (PLS-DA), to detect stress across various growth stages. Furthermore, severity levels were objectively defined using photoreflectance indices and principal component analysis (PCA) data visualizations, which enabled consistent and reliable classification of stress responses in both individual cultivars and combined datasets. All models achieved high classification accuracy (90–98%) on independent test sets. The application of the Successive Projections Algorithm (SPA) for variable selection significantly reduced the number of wavelengths required for robust stress classification, with SPA-PLS-DA models maintaining high accuracy (90–96%) using only a subset of informative bands. Furthermore, SPA-PLS-DA-based chemical imaging enabled spatial mapping of stress severity within plant tissues, providing early, non-invasive insights into physiological and biochemical status. These findings highlight the potential of integrating hyperspectral imaging and machine learning for precise, real-time crop monitoring, thereby contributing to sustainable agricultural management and reduced yield losses.

## 1. Introduction

Potato (*Solanum tuberosum* L.) and sweet potato (*Ipomoea batatas* L. Lam.) are among the most widely consumed staple crops, valued for their high content of starch, dietary fiber, and essential nutrients [1,2]. In terms of global production, potatoes rank as the fourth most cultivated carbohydrate-rich crop, after rice, wheat, and maize, while sweet potatoes rank as the sixth, underscoring their vital role in global food security [2,3]. Despite their agricultural and economic significance, these crops are highly susceptible to seasonal biotic and abiotic environmental stressors that significantly impact their yield and quality. In Korea, potatoes are typically transplanted in late March and harvested by early June, making them susceptible to heat and water stress during critical growth stages. Similarly, sweet potatoes, planted in mid-May and harvested in late September, often face drought and excessive water stress, which compromises productivity and leads to yield loss [4,5].

Drought, heat, and excessive water stress disrupt key developmental stages, limiting tuber formation and reducing productivity [6,7,8]. Physiological effects such as reduced photosynthesis, impaired leaf expansion, and premature senescence further exacerbate yield losses [9,10,11]. At the same time, excessive water stress increases the frequency of flooding due to climate change [12], resulting in oxygen deprivation in the root zone [13] and enhancing disease susceptibility and damage [12]. Heat stress delays sprout emergence, reduces stolon formation, and impairs tuber initiation and bulking. These disruptions, along with altered photosynthesis and carbohydrate partitioning, ultimately reduce starch accumulation, thereby affecting tuber yield and quality [13,14]. Given the sequential nature of potato growth, stress at any stage can accumulate, compounding its impact on subsequent growth phases. Therefore, continuous monitoring of plant responses to environmental stressors is essential for mitigating their effects, optimizing resource management, and supporting the resilience of cropping systems and food security.

Advancements in vision sensors have revolutionized plant stress detection, offering precise, non-destructive monitoring compared to traditional methods. Conventionally, plant stress identification relied on visual inspection of symptoms such as leaf discoloration, wilting, and stunted growth [15], as well as manual measurements of turgor pressure [16] and leaf water content [17,18]. However, these approaches are labor-intensive, subjective, and inefficient. In contrast, vision sensors enable the automated, objective detection of plant stress by capturing morphological and physiological changes and analyzing stress responses through advanced machine learning and deep learning techniques [15]. Color imaging sensors, widely used due to their affordability and speed, have been successfully applied for detecting plant stress, including abiotic stress, and estimating water stress [19], temperature stress (Basavaraj R. Amogi, 2024), salt stress (Huaichuan Yang, 2025), and biotic stress, including root rot disease identification [20], and rust disease monitoring in leaves [21]. However, their reliance on surface-level information limits their ability to detect early-stage plant stress before visible symptoms appear [22].

Integrating advanced imaging technologies into non-destructive sensors (NDS) has significantly enhanced plant health assessment by detecting stress-induced chemical alterations before symptoms become visible. Spectroscopic imaging techniques, such as line scan and snapshot HSI systems, are non-invasive methods that combine imaging and spectroscopy to record both the physical attributes (shape, size, appearance, and color) and biochemical composition of crops through spectral analysis [23,24]. Specifically, comprehensive reviews have documented both its strengths, such as huge spectral resolution and the ability to capture subtle biochemical changes, and its limitations, including high data dimensionality and the need for robust calibration protocols [23,25].

In this study, potato and sweet potato plants were grown from the initial onset of seasonal stress through to crop production, capturing the full impact of environmental stressors throughout their development. The experiment was specifically designed to simulate natural seasonal temperature variations in South Korea, exposing plants to key ecological stresses, including seasonal drought, heat, and excessive water, thereby reflecting the real conditions these crops face. Korea’s distinct four seasons bring vulnerabilities such as spring droughts and summer monsoons, with future climate projections indicating increased drought duration, higher temperatures, and elevated rainfall [26]. These climatic changes are expected to negatively impact both the yield and quality of potato crops and sweet potato crops [27]. Given their typical growing periods, potatoes from late March to early June and sweet potatoes from mid-May to late September, these crops face significant risks from water-related and temperature stresses.

Detecting plant seasonal stress at various levels is critical for precise crop management and sustainable agriculture. Early identification of stress symptoms, whether induced by drought, excessive water, or temperature fluctuations, enables timely interventions such as optimized irrigation scheduling, nutrient management, or adaptive breeding strategies. To achieve this, comprehensive experiments were conducted throughout the complete growth cycles of potato and sweet potato plants, applying drought, elevated temperature, and excessive water stress at key phenological stages ranging from emergence through tuber bulking to senescence. Utilizing a mobile VNIR hyperspectral platform, whole-plant reflectance profiles were captured under each stress condition, allowing dynamic updates of baselines and thresholds during plant development. Photoreflectance indices and PCA were used to visualize the effects of stressors and to categorize data according to stress severity levels. The SPA was employed to identify the most informative wavebands for distinguishing between stress levels. Machine-learning classifiers, including SVM, LDA, and PLS-DA, were then trained on these optimized spectra to classify plant stress throughout the growth cycle accurately.

The specific objective of this study was to develop and calibrate a life-cycle–agnostic VNIR hyperspectral imaging platform for greenhouse monitoring of potato and sweet potato under drought, heat, and excess-water stress, and to: (i) implement stage-aware spectral calibration with adaptive decision thresholds valid from emergence to senescence; (ii) define stress-severity levels directly from reflectance signatures; (iii) design and benchmark machine-learning classifiers (PLS-DA, LDA, SVM) to identify the most effective model for severity discrimination; (iv) determine a compact, physiologically meaningful wavelength set via SPA; (v) integrate spectral classification with pixel-level severity mapping for canopy-scale visualization. Together, these objectives advance hyperspectral imaging toward a scalable, transferable framework for real-time, non-destructive stress monitoring and management in staple crops under climate variability.

## 2. Results

### 2.1. Spectral Characteristics of Environmentally Stressed Plants

**Figure 1:**
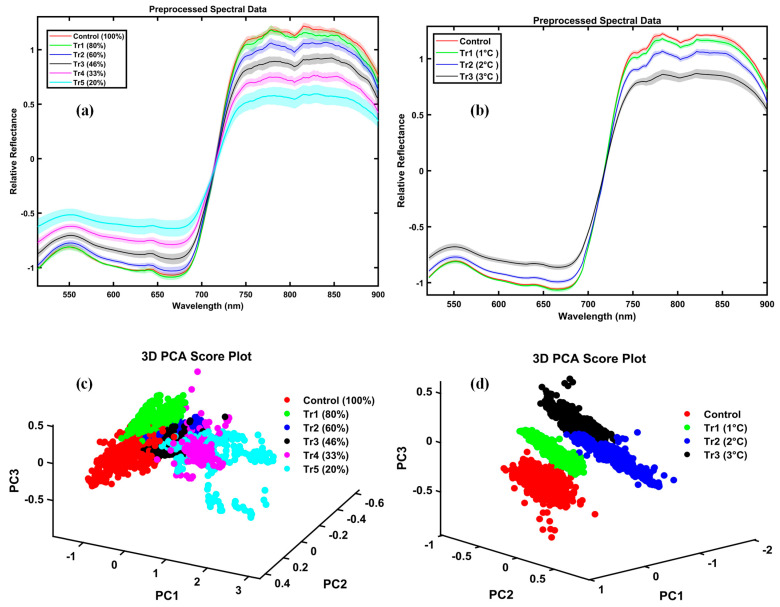
Mean SNV-preprocessed spectral reflectance curves and PCA score plots of potato plants under drought and temperature stress. (**a**) Reflectance spectra for drought stress treatments and (**b**) temperature stress treatments. Solid lines represent mean reflectance values, while shaded regions indicate ± standard deviation for each group. Treatments are color-coded as shown in the legends. (**c**) Three-dimensional PCA score plot for drought stress and (**d**) temperature stress, where each point corresponds to an individual spectrum. Clustering patterns highlight a clear separation among treatment groups, consistent with spectral differences observed in panels (**a**,**b**).

Figure 1 presents the mean spectral reflectance of drought- and temperature-stressed potato plants, preprocessed using the Standard Normal Variate (SNV) method, with shaded regions representing the standard deviations for the control and treated groups. Spectral preprocessing significantly enhanced data consistency by effectively removing unwanted spectral variations caused by baseline shifts, scattering effects, and path-length differences. Panel (a) shows the progressive spectral changes under drought stress, with reflectance decreasing from full irrigation (100%) to severe deficit (20%). Control plants exhibit lower reflectance in the visible range (400–730 nm) compared to plants subjected to 60% or less irrigation, which show elevated reflectance. Conversely, in the near-infrared (NIR) region (730–900 nm), the preprocessed spectral data revealed higher reflectance for healthy plants compared to stressed plants. Panel (b) depicts the effects of temperature stress. Higher temperatures (+2 °C and +3 °C) accelerated the increase in reflectance across both visible and NIR wavelengths. In contrast, control and mildly stressed (+1 °C) plants exhibited nearly identical spectral profiles. In healthy potato leaves, pigments such as chlorophyll and carotenoids efficiently absorb visible light, particularly in the blue (~450 nm) and red (~670 nm) regions, resulting in lower reflectance.

To further enhance data interpretation, panels (c) and (d) of Figure 1 present the distribution of spectral data points using PCA for the drought and temperature stress datasets, respectively. In the drought stress analysis, the first principal component (PC1) captures 85.4% of the total variance, with the second component (PC2) accounting for an additional 5.5%. Together, these two components account for 90.9% of the variance, facilitating a clear separation among different irrigation treatments. Similarly, for temperature stress, PCA reveals distinct clustering of treatment groups relative to the control, with PC1 and PC2 accounting for 53% and 15.9% of the variance, respectively, for a combined total of 68.9%. These visualizations provide intuitive insights into the distribution and separability of the data under various stress conditions.

**Figure 2:**
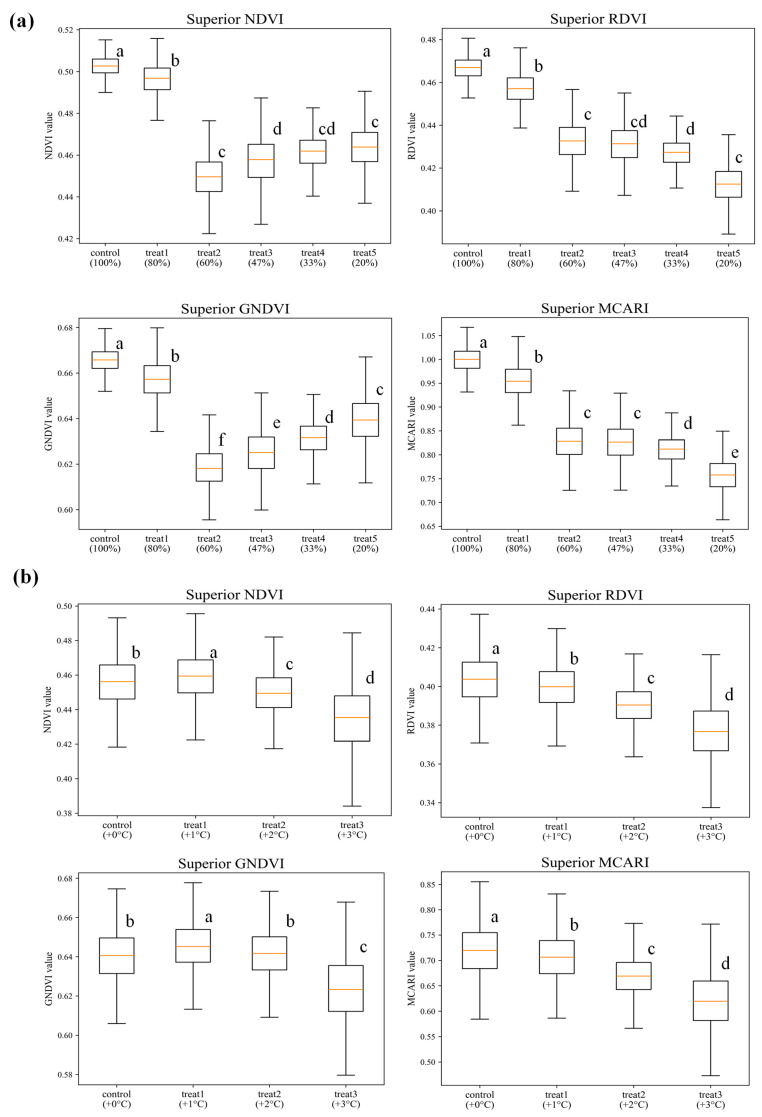
Vegetation indices derived from potato plants subjected to drought stress (**a**) (top four panels) and temperature stress (**b**) (bottom four panels). Shown are boxplots of NDVI, RDVI, GNDVI, and MCARI for each stress treatment. In each plot, the central line represents the median, boxes indicate interquartile ranges, and whiskers show data spread. Different lowercase letters above the boxes indicate statistically significant differences among treatments (*p* < 0.05) based on one-way ANOVA followed by Duncan’s multiple range test.

Furthermore, vegetation indices (NDVI, RDVI, GNDVI, MCARI) derived from the hyperspectral data closely mirrored the spectral patterns observed in Figure 2, underscoring their robustness in capturing stress-induced physiological and biochemical changes. In drought-stressed potato plants (Figure 2a), index values remained stable under control (100%) and moderate (80%) irrigation but declined sharply at 60% and below, effectively marking the onset of severe stress. Notably, this decline provided a more precise boundary than spectral profiles alone, which often made it difficult to distinguish between transitional stages. A slight rebound at more severe irrigation deficits suggested the presence of possible acclimation mechanisms to prolonged drought. Under temperature stress (Figure 2b), a similar trajectory was observed, with plants at +2 °C and +3 °C showing marked reductions relative to the control and +1 °C treatments, again providing sharper separation of severity levels. Collectively, these results highlight vegetation indices as a valuable complement to spectral and PCA, enabling physiologically interpretable thresholds that improve the delineation of control, early-stage, and severe stress categories. Statistically significant differences among indices of each treatment were evaluated using one-way ANOVA, followed by Duncan’s multiple range test (DMRT) at α = 0.05, after verifying normality of residuals (Shapiro–Wilk) and homogeneity of variances (Levene’s test). Sample size (n) denotes the number of plant-level images per treatment at each growing stage. Overall, the concordance between spectral reflectance and vegetation indices supports the use of hyperspectral imaging as a non-destructive tool for monitoring subtle physiological and structural changes in response to abiotic stress.

### 2.2. Spectral-Based Classification of Stress Severity Level

Based on the analysis of spectral reflectance and vegetation indices in Figure 1 and Figure 2, respectively, potato plants were systematically grouped into stress severity categories that reflect their underlying physiological and biochemical status. For drought treatments, plants receiving 80% irrigation showed spectral and index values similar to those of the controls, indicating minimal disruption and classifying them as experiencing early-stage drought stress. In contrast, plants irrigated at 60% or less exhibited pronounced increases in visible reflectance (400–730 nm) and near-infrared reflectance (730–900 nm), corresponding to pigment loss, tissue damage, and moisture depletion. These spectral shifts were closely associated with significant declines in NDVI, RDVI, GNDVI, and MCARI indices, confirming severe drought stress.

**Figure 3:**
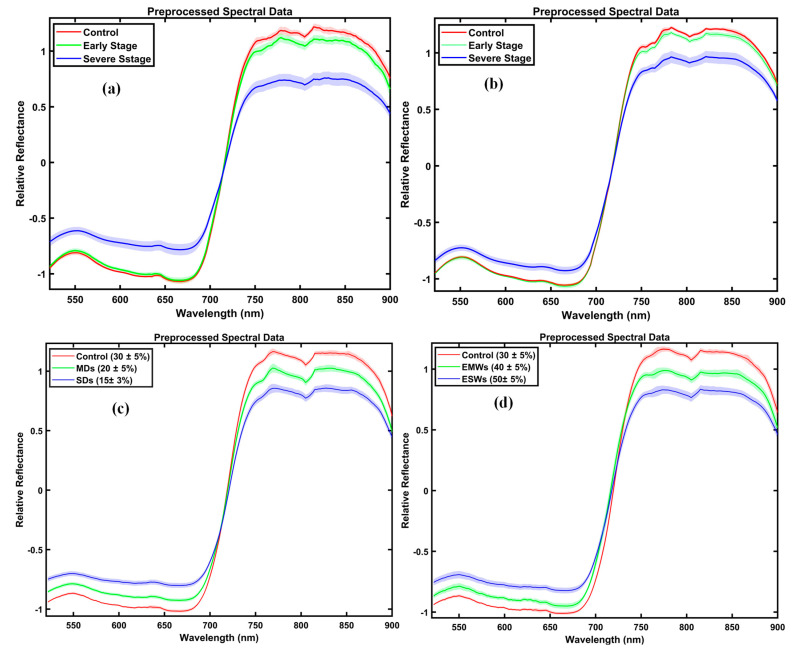
Mean SNV-preprocessed spectral reflectance profiles of potato and sweet potato plants under different abiotic stress conditions. (**a**) Potato plants under drought stress were grouped into three categories: control, early-stage (80% irrigation), and severe (≤60% irrigation). (**b**) Potato under temperature stress, with early-stage defined by +1 °C, severe stress by +2 °C, and +3 °C. (**c**) Sweet potato under drought stress categorized by soil moisture thresholds. (**d**) Sweet potato under excessive water stress, separated into moderate (EMWs) and severe (ESWs) stress levels. Solid lines show mean reflectance, shaded areas indicate ± standard deviation, and treatment groups are defined in the legends.

As illustrated in (Figure 3a), this stratification allowed for clear grouping of drought-stressed potato plants into control, early-stage (80% irrigation), and severe (≤60% irrigation) categories. PCA (Figure 3a) further supports these divisions, with control and early-stage groups clustering closely, while severe stress groups are more dispersed due to advanced physiological and biochemical damage. For temperature stress (Figure 3b), plants exposed to a mild increase (+1 °C) exhibited negligible spectral deviation from controls, validating their classification as early-stage temperature stress. Greater temperature elevations (+2 °C and +3 °C) resulted in marked increases in both visible and NIR reflectance, indicating more substantial physiological disruption. PCA (Figure 3d) distinctly separates these groups in multivariate space, supporting classification into control, early-stage (+1 °C), and severe stress levels (+2 °C and +3 °C).

For sweet potato plants, stress severity was determined using specific soil moisture thresholds, rather than initially relying on spectral signatures, vegetative indices, and PCA data visualization. Panels (c) and (d) display the average spectral reflectance for drought and excessive water treatments, measured over 11 weeks. Under drought stress (Panel c), both visible (520–700 nm) and near-infrared (750–900 nm) reflectance increased significantly, indicating the breakdown of pigments (such as chlorophyll and carotenoids) and damage to the leaf structure caused by a long-term water shortage. Notably, sweet potatoes exposed to early-stage drought already showed higher reflectance than controls, likely due to the cumulative effects of ongoing moderate stress. For excessive water stress (Panel d), spectral changes were more subtle but consistently observed in the same wavelength regions. As soil moisture increased from moderate (EMWs; 40 ± 5%) to severe (ESWs; ≥50 ± 5%), reflectance also rose slightly, reflecting a decline in pigment concentration and changes in leaf internal structure due to persistent waterlogging. High soil moisture limited oxygen for roots, disrupted nutrient and water uptake, and increased the risk of root diseases.

Overall, the distinct spectral separation observed among healthy, moderately stressed, and severely stressed plants underscores the strong discriminative ability of hyperspectral imaging to detect physiological states across stress gradients. To ensure robust development of the classification model, the spectral datasets were systematically partitioned into calibration (60%), validation (20%), and independent test sets (20%), as summarized in Table 1.

In addition, the SPA was applied to identify the most informative wavelengths. Depending on cultivar and stress treatment, SPA selected between 21 and 32 bands, representing a substantial dimensionality reduction while retaining predictive information. For example, under drought stress, 23 optimal wavelengths were identified for Jopung, 24 for Superior, and 27 for the combined dataset. Under temperature stress, 31 and 32 bands were selected for Jopung and Superior, respectively, with 25 bands shared in the combined model. For sweet potato (Jinyulmi), SPA identified 32 informative bands under drought stress and 21 under excessive water stress.

Together, these SPA-selected subsets, combined with the complete spectral datasets, provided a strong foundation for subsequent classification analyses and for comparing model performance with respect to both accuracy and computational efficiency.

### 2.3. Performance Assessments of Classification Models: LDA, SVM, PLS-DA, and SPA-PLS-DA

Table 2 presents a comparative analysis of the classification accuracy of LDA, SVM, and PLS-DA models developed for hyperspectral data from the Jopung and Superior potato cultivars. These models were constructed to distinguish control, early-stage, and severe stress classes using both raw and preprocessed spectra. Initially, all models were trained on the complete spectral range of 195 bands to establish baseline performance. Subsequently, the SPA was employed to identify the most informative wavelengths from high-performing, preprocessed data. This approach substantially reduced data dimensionality and spectral redundancy, thereby enhancing computational efficiency without sacrificing classification accuracy.

Across all models, classification accuracies for calibration, validation, and test sets remained consistently high. For Jopung under drought stress, PLS-DA with Savitzky–Golay 2nd derivative preprocessing achieved a test accuracy of 99.4%, closely followed by LDA and SVM, both of which exceeded 95%. Using 23 SPA-selected bands, PLS-DA retained high accuracy (98.2%), demonstrating the method’s effectiveness in preserving critical spectral information. For the Superior cultivar, PLS-DA achieved 96.7% test accuracy using raw spectra and 94.1% with 24 SPA-selected bands, balancing dimensionality reduction with a minor loss in predictive power. Notably, SPA identified 10 shared critical bands between Jopung and Superior for drought classification. Combining data from both cultivars into a unified model yielded slightly reduced but still robust test accuracies (94.18% for the full spectrum and 91.04% for SPA-selected), reflecting increased spectral variability.

Under temperature stress, PLS-DA again outperformed other models, achieving up to 99.3% accuracy (Superior, range normalization), with all models exceeding 95% for most datasets. SPA-selected bands (31–32) for temperature stress maintained high accuracy, with Jopung and Superior both around 96%, and the combined dataset achieving 93.99% (full spectrum) and 91.50% (25 bands). Many SPA-selected bands overlapped across cultivars, indicating robust feature selection.

Across all stress classification tasks and model variations in Table 3, the test set performance metrics demonstrated remarkable consistency and reliability. Precision, recall, and F1-scores for all models ranged from 0.89 to 0.99, while AUC values consistently fell between 0.97 and 1.00. The highest-performing models, most notably PLS-DA, achieved precision, recall, and F1-scores exceeding 0.98, with AUC values approaching 0.999. Even after substantial dimensionality reduction through SPA band selection, these metrics remained robust, with the lower end of precision, recall, and F1-score values rarely dipping below 0.90.

Building upon the insights gained from classifying drought and temperature stress in Jopung and Superior potato varieties (Table 2), further evaluation was conducted on the sweet potato variety Jinyulmi under drought and excessive water stress conditions (Table 3). The performance trends observed in potato plants were primarily reflected in the sweet potato data, with all three models, PLS-DA, SVM, and LDA, demonstrating strong classification capabilities across different stress levels. Notably, PLS-DA consistently outperformed the other models, attaining test accuracies up to 99.45% for drought stress and 97.8% for excessive water stress when utilizing the full spectral dataset. Although SPA-based band selection (32 bands for drought, 21 bands for excessive water) resulted in a slight decrease in accuracy (to 95.23% and 90.53%, respectively), it substantially reduced model complexity and computation time.

**Figure 4:**
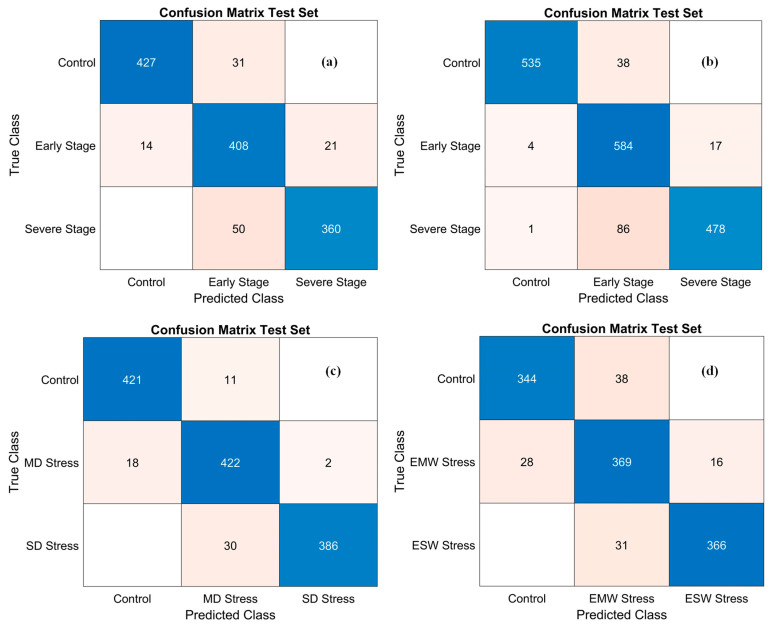
Confusion matrices illustrating the classification performance of SPA-PLSDA models on the test sets. (**a**) Potato under drought stress, (**b**) potato under temperature stress, (**c**) sweet potato under drought stress, and (**d**) sweet potato under excessive water stress. The X-axis represents predicted class labels, and the Y-axis represents true class labels. Color intensity corresponds to the number of samples in each classification outcome.

To further evaluate the discriminative power of the SPA-PLSDA models, confusion matrices were generated, with predicted class labels plotted on the X-axis and true class labels on the Y-axis. As shown in Figure 4, panels (a) and (b) present the validation results for the combined Jopung and Superior potato cultivars under drought and temperature stress, respectively. These matrices reveal strong classification performance, with only minor misclassifications predominantly occurring between adjacent stress categories, most notably between early-stage and severe stress groups. Such errors likely result from subtle spectral similarities during transitional phases, where physiological differences are less distinct.

Panels (c) and (d) display the confusion matrices for sweet potato under drought and excessive water stress; the models demonstrated high predictive accuracy. However, some confusion was observed between the control and moderately stressed (MS) categories, as well as between mild and severe drought (MD and SD) or moderate and severe excessive water stress (EMW and ESW). These misclassifications are attributable to greater physiological variability in sweet potato and the extended duration of sampling, which captured both the onset and progression of stress symptoms. For excessive water stress, minor misclassifications occurred between the Control and EMW groups, particularly in early sampling weeks when physiological differences were minimal. Notably, no samples were misclassified between the Control and severe stress categories in either drought or excessive water treatments, underscoring the models’ robust ability to distinguish healthy from severely stressed plants based on spectral signatures.

### 2.4. Imaging-Based Visualization of Plant Stress Responses

Building on the classification models optimized for each cultivar and stress type, PLS-DA consistently achieved the highest accuracy across all datasets. For image-based visualization of plant stress, hyperspectral images were preprocessed using the same optimal methods that yielded the best classification results: range normalization for potato drought stress, raw spectra for potato temperature stress, MSC for sweet potato drought stress, and raw spectra for sweet potato excessive water stress. PLS-DA response images were then generated using these tailored preprocessing approaches, ensuring that visualization was entirely consistent with model development and performance as detailed in Table 2 and Table 3.

**Figure 5:**
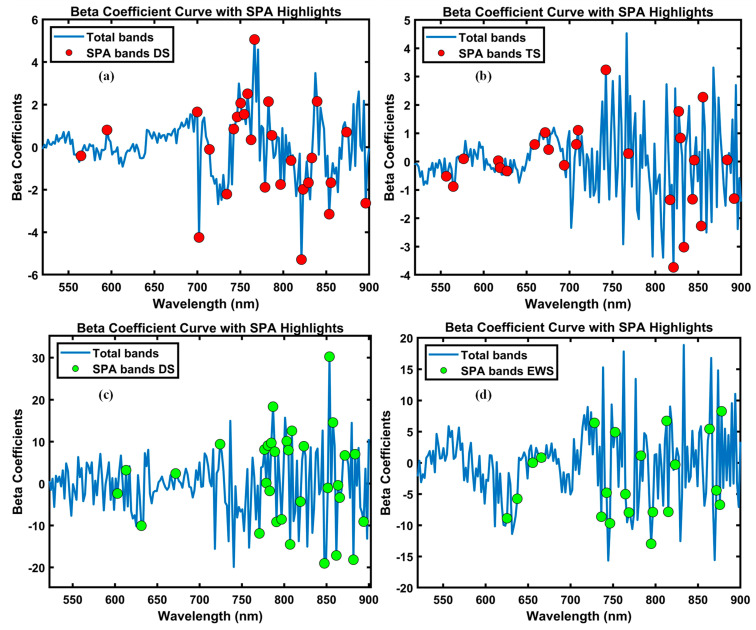
Beta coefficient curves from SPA-PLSDA models highlighting informative wavelengths for stress detection. (**a**) Potato under drought stress, (**b**) potato under temperature stress, (**c**) sweet potato under drought stress, and (**d**) sweet potato under excessive water stress. Blue lines represent regression coefficients across the full spectral range, while colored circles indicate bands selected by the SPA.

Figure 5 illustrates these beta coefficient profiles of total and selected bands for both potato and sweet potato plants under various abiotic stresses. Panels (a) and (b) present the drought and temperature stress responses in potato cultivars, with SPA-selected bands combined spectral markers indicated by red dots, respectively. Panels (c) and (d) show drought and excessive water stress in sweet potato plants, with selected bands indicated by green dots. Prominent features in the visible region (520–740 nm) are linked to alterations in photosynthetic pigments, with chlorophyll-related signatures evident near 556.5 nm and along the red-edge around 724.2 nm. Moreover, shifts within the red-edge interval (690–740 nm) closely track chlorophyll loss, aligning with previous reports [28,29]. In parallel, changes across the NIR window (740–900 nm) correspond to variation in internal cellular structure and water status, reflecting stress-induced physiological damage [30]. Notably, our spectra exhibited the same pattern: distinct inflections near ~740 nm and ~840 nm, wavelengths commonly attributed to water absorption bands [31,32]. These assignments are consistent with the SPA-PLS-DA coefficient peaks obtained in this study. The peaks detected at approximately 742.4 nm and 841.8 nm are likely attributable to the overtone absorption of water, consistent with earlier descriptions.

Consequently, the beta coefficients derived from the SPA-PLSDA models, corresponding to each preprocessing method for potato and sweet potato plants, were applied pixel-wise to hyperspectral images that had been preprocessed identically to their respective models. As detailed in Table 1, the selected bands included 27 for drought stress in the combined Jopung and Superior SPA-PLSDA model, 25 for temperature stress in the combined Jopung and Superior model, 32 for drought stress in Jinyulmi, and 21 for excessive water stress in Jinyulmi. These bands were subsequently employed to generate chemical images, offering spatially resolved visualization of stress-induced physiological variations within the plants.

**Figure 6:**
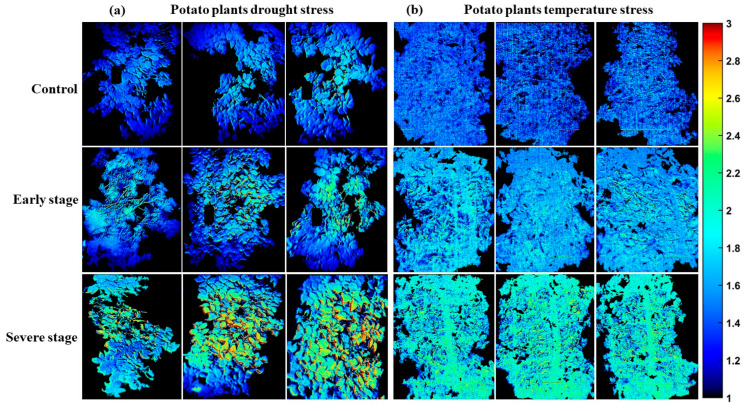
PLS-DA response images of potato plants under (**a**) drought stress and (**b**) temperature stress. The color scale, ranging from blue to yellow/green, indicates increasing stress severity, with blue corresponding to healthier tissues and yellow/green highlighting regions of pigment loss and physiological damage.

**Figure 7:**
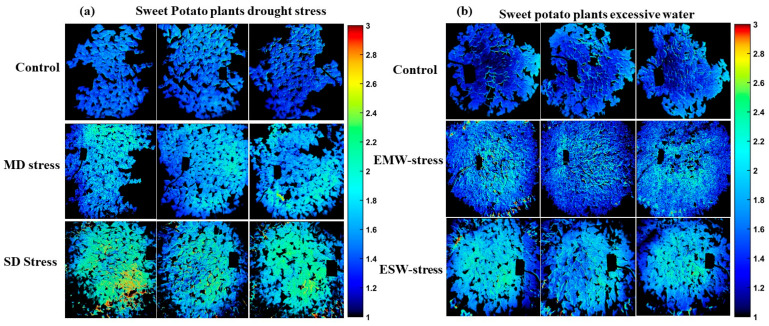
PLS-DA response images of sweet potato plants under (**a**) drought stress and (**b**) excessive water stress. The color scale, ranging from blue to yellow/green, indicates increasing stress severity, where blue corresponds to healthier tissues and yellow/green highlights pigment loss and physiological decline.

Figure 6 shows PLS-DA response images for potato plants under drought (Panel a) and temperature stress (Panel b). In (a), control, early-stage, and severe drought stress are shown in consecutive rows, with early-stage plants closely resembling controls in both spectral and chemical characteristics, indicating a milder physiological impact. Panel (b) illustrates distinct differences in chemical composition among the temperature treatments. Across Figure 6 and Figure 7, the blue-to-yellow/green color gradient represents increasing stress severity, where blue indicates healthier tissue with higher chemical content, and yellow/green marks regions of pigment loss and physiological deterioration. A threshold of 0.5 was used to define class boundaries.

Similarly, Figure 7 depicts sweet potato plants under drought and excessive water stress. Panel (a) reveals a progressive increase in yellow-green hues from control to moderate (MD) and severe drought (SD) stress, again emphasizing pigment loss and moisture depletion, especially in top leaves. In contrast, panel (b) shows excessive water stress (EMW and ESW), where chemical changes are subtler and more evenly distributed throughout the canopy, likely due to root zone saturation affecting the entire plant. These visualizations confirm that drought stress induces localized, progressive deterioration, whereas excessive water stress causes broader but less intense physiological alterations.

## 3. Discussions

A comprehensive, life–cycle–based evaluation of crop seasonal stress responses is essential for developing precise and crop management strategies, particularly for root crops such as potatoes and sweet potatoes, which face diverse abiotic challenges throughout their development. This study establishes a robust spectral framework for rapid, non-destructive classification of seasonal abiotic stress in potato and sweet potato plants. By leveraging hyperspectral reflectance data and integrating PCA with machine learning models, including PLS-DA, LDA, and SVM, we achieved accurate discrimination of stress severity across drought, temperature, and excessive water conditions.

HSI effectively captured the coupled biochemical and physiological responses of potato and sweet potato to drought, temperature, and excessive water stress. In healthy leaves, chlorophylls and carotenoids strongly absorb light in the blue (450 nm) and red (670 nm) ranges, resulting in characteristically low reflectance in the visible range [31]. By contrast, the NIR region (730–900 nm) is characterized by significant internal scattering and influenced by leaf water status, producing a broad zone of high reflectance typical of unstressed leaves [30]. Under stress, pigment degradation and tissue disruption altered the spectral profile, increasing reflectance in the visible range through reduced pigment absorption and decreasing NIR reflectance due to loss of structural integrity and water content. These spectral alterations were most evident under severe drought (≤60% irrigation) and elevated temperature treatments (+2 °C and +3 °C), whereas mildly stressed plants (+1 °C or 80% irrigation) closely resembled the controls, indicating minimal early physiological disturbance.

Moreover, multivariate analysis and vegetation indices revealed patterns consistent with the spectral interpretation. PCA provided a clear separation of the treatment groups, explaining 90.9% of the variance under drought and 68.9% under temperature stress, thereby confirming that hyperspectral data effectively captured the progression of stress. Complementing this, vegetation indices (NDVI, RDVI, GNDVI, MCARI) exhibited significant declines as stress intensified. By combining reflectance information from the red and near-infrared regions, these indices provided integrative measures of canopy function, including pigment status, structural integrity, and photosynthetic activity [33]. For example, the NDVI is widely used to evaluate chlorophyll and nitrogen levels in plants [34]. For drought stress in potato plants, NDVI exhibited a distinct threshold: control plants clustered with the 80% irrigation treatment, while values dropped sharply at 60% and below, allowing for classification into control, early-stage, and severe stress groups. Similar trajectories were observed for RDVI, GNDVI, and MCARI, all of which declined progressively with increasing stress. Under heat stress, a comparable trend emerged, with plants at +2 °C and +3 °C showing marked reductions relative to the control and +1 °C treatments. Together, these multivariate and index-based results reinforce that hyperspectral data encode stress severity in both the visible and red-edge regions, where pigment loss is most evident, and in the NIR region, where dehydration and tissue disruption are expressed. The alignments between PCA clustering and vegetation indices underscore the robustness of this framework for defining stress boundaries in potato plants.

In continuation of the spectral and index-based interpretation, the classification analysis confirmed that hyperspectral data can accurately delineate stress severity across cultivars and stress conditions. Among the tested algorithms, PLS-DA consistently outperformed LDA and SVM, particularly when combined with appropriate preprocessing, reflecting its strength in maximizing covariance between spectral features and categorical labels in high-dimensional datasets. Model performance remained robust across calibration, validation, and independent test sets, with accuracies frequently exceeding 95% and AUC values approaching unity. When data from multiple cultivars were integrated into a single model, accuracies declined modestly relative to single-cultivar models due to increased biological variability; however, performance still exceeded 91%, underscoring the robustness of the framework for multi-cultivar stress monitoring.

Following the evaluation of full-spectrum models, SPA was applied to identify the most informative wavelengths, reducing the input from 195 bands to 21–32 while retaining the essential physiological information. This dimensionality reduction enhanced computational efficiency and interpretability without compromising diagnostic accuracy. The subsequent confusion matrix analyses further clarified model behavior, showing that misclassifications were confined mainly to adjacent categories of early and severe stress. Notably, no misclassification occurred between control and severe classes, demonstrating the model’s reliability in correctly identifying high-risk plants, which is essential for field-level stress detection and management. Slightly greater confusion is observed in sweet potato, particularly under excessive water stress, likely reflecting broader physiological variability and the diffuse effects of waterlogging.

The models optimized with SPA-selected bands were further applied for imaging-based visualization, enabling classification results to be directly translated into spatial maps of plant stress. Beta-coefficient profiles consistently highlighted spectral features in the visible and red-edge regions (around 556.5 nm and 724 nm), corresponding to chlorophyll absorption, consistent with earlier findings [28,29,35]. Additionally, NIR features (740–900 nm) linked to internal leaf structure and water status [30,36]. In this study, the most prominent peaks were detected at 556.5 nm, 724 nm, 742.4 nm, and 841.8 nm. Specifically, the peaks at 742.4 nm and 841.8 nm are associated with water-related overtone absorption, while those near 556.5 nm and 724 nm reflect chlorophyll dynamics and red-edge shifts. Pixel-wise application of the coefficients generated chemical maps that localized stress expression within the canopy. In potato plants, drought stress affected the entire canopy but was more pronounced in newly emerging apical leaves, whereas temperature stress produced a more uniform response across the whole canopy. In sweet potato, drought led to progressive yellow-green patterns reflecting pigment loss and water depletion, particularly in the upper canopy. In contrast, excessive water stress caused subtler but more widespread alterations, consistent with root-zone oxygen limitation and disrupted nutrient uptake, and exacerbated physiological stress and tissue damage [12]. These spatially resolved maps validated the classification results and offered actionable insight into the development and distribution of stress symptoms across plant organs and stress conditions.

Collectively, the convergence of (i) mechanistic spectral changes, (ii) PCA and index trends, (iii) consistently high model performance, (iv) efficient SPA-based band reduction, and (v) physiologically interpretable stress maps establishes hyperspectral imaging, coupled with PLS-DA and targeted wavelength selection, as a robust non-destructive framework for early stress detection. The reduced band sets highlight the potential for compact, cost-effective multispectral solutions, for a clear separation between control and severe classes ensures reliability for field detection. Notably, bands around 556 nm and 724 nm (corresponding to chlorophyll absorption) and around 742–743 nm and 840–842 nm (water overtones) emerged as physiologically informative, aligning with the spectral coverage of existing multispectral sensors. Together, these outcomes point toward practical deployment of real-time, high-throughput stress monitoring to support precision irrigation, targeted interventions, and climate-resilient crop management.

Despite the strengths of this study, several limitations remain. First, all experiments were conducted under greenhouse conditions, where illumination and microclimate were relatively stable; thus, field deployment will require explicit handling of variable lighting, canopy shading, and soil background effects through radiometric normalization or reflectance standards [25]. Second, while three abiotic stresses (drought, temperature, and excessive water) were examined, real agricultural systems often involve simultaneous interactions with other abiotic factors and biotic stresses such as pathogens and pests, which were not addressed here and may produce overlapping spectral signatures. Third, although SPA effectively reduced dimensionality by identifying 21–32 key bands, hyperspectral imaging still produces large datasets that are computationally intensive; scaling this approach to field or UAV platforms will require cost-effective multispectral sensors and on-edge analytics. Finally, the high accuracies achieved in this work benefited from controlled conditions and single-crop canopies; in field settings, additional variability due to mixed pixels, phenological changes, and sensor drift may reduce performance unless models are carefully adapted and validated across seasons, cultivars, and devices.

For future investigations, three directions appear most impactful: rigorous field validation across seasons, cultivars, and management regimes, with domain-adaptation strategies to improve transferability; sensor fusion to stabilize classification under confounded stress environments; and deployment of SPA-guided, lean multispectral platforms with embedded PLS-DA inference for real-time decisions from tractor, boom, or UAV. Taken together, the physiology-grounded spectra, compact classifiers, and canopy-level visualization demonstrated here provide a scalable, non-destructive framework for early, objective stress surveillance in potato, sweet potato, and other staple crops.

## 4. Materials and Methods

### 4.1. Experimental Design

In this study, three commercially important cultivars were selected: Jopung and Superior for potato, and Jinyulmi for sweet potato. Seasonal growth and key abiotic stresses, including drought, temperature, and excessive water stressors, were carefully designed and imposed under controlled greenhouse conditions, ensuring consistent and realistic stress exposure for each cultivar. Potato cultivation was conducted from April to May 2024 at the Rural Development Administration, National Institute of Crop Science (RDA-NICS) in Jeonju, South Korea (35.8437° N, 127.0475° E), located 29 m above sea level. Simultaneously, drought and excessive water stress experiments for sweet potatoes were conducted at Gyeongsang National University, Naedong Campus, Jinju-si, Gyeongsangnam-do, South Korea (35.1548° N, 128.0856° E), at an elevation of 30 m, from 20 June to 4 September 2024, spanning 119 days. These sites and experimental periods were strategically selected to align with the natural growing seasons of each crop in South Korea. This design enabled a detailed assessment of how each cultivar responds to environmental stresses under conditions that reflect those encountered in local agricultural practice and anticipated climate change scenarios. Table 4 summarizes the experimental design, including the number of greenhouses, treatment levels, sampling regimes, and the total hyperspectral images collected per treatment.

Drought-exposed potato plants were grown in separate greenhouses (length 25 m, width 2 m) and two greenhouses (GH), one per cultivar. A drip irrigation system was used to regulate the water supply with high precision. Experimental plants were assigned to six (rows) treatment groups, each receiving a specific proportion of irrigation water. The control group received 100% of the recommended water amount, equivalent to 8.3 L per row daily, delivered over a 15 min irrigation period. The optimal irrigation level was defined based on the optimal soil volumetric water content (SVWC) for potato growth (30 to 35%), which has been shown to support high potato yield with efficient water use [7,37,38,39]. Throughout the experiment, SVWC was continuously monitored and maintained at 30 ± 3% using TEROS 12 sensors (METER Group, Pullman, WA, USA), with measurements taken daily. In contrast, the remaining groups were subjected to progressively reduced irrigation levels: 80% (6.6 L over 12 min), 60% (5.0 L over 9 min), 47% (3.8 L over 7 min), 33% (2.8 L over 5 min), and 20% (1.7 L over 3 min). Table 4, second row, provides a summary of the experimental design, including the number of hyperspectral images collected weekly and throughout the entire drought-exposed experimental plants.

The heat stress experiment was conducted in three greenhouses, each applying heat stress simultaneously to assess plant responses under varying temperature conditions throughout their development. Ambient air temperature was continuously monitored by calibrated sensors placed outside each greenhouse throughout the experiment, measuring temperature every hour, which ranged from a minimum of 2.2 °C to a maximum of 31 °C. These sensors were synchronized to regulate the temperature of the greenhouse control plants in accordance with external environmental conditions. The three greenhouses replicated the same treatments (Tr), each maintaining temperature regimes at +1 °C (Tr 1), +2 °C (Tr 2), and +3 °C (Tr 3) above the continuously recorded outdoor temperature. Each greenhouse contained one row divided into four zones corresponding to the control and the three elevated temperature treatments described earlier. Temperature data were recorded continuously throughout the entire plant life cycle, as previously described, allowing for a comprehensive assessment of the effects of temperature stress at each developmental stage.

**Figure 8:**
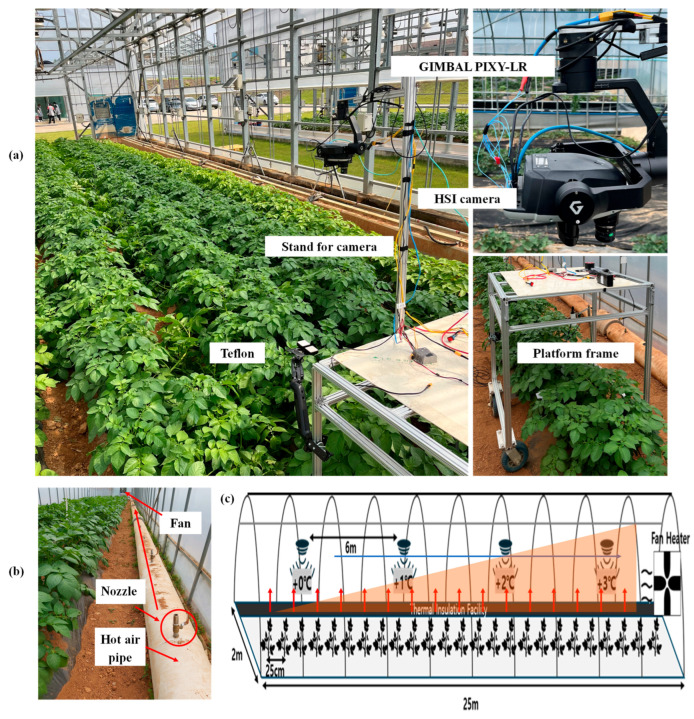
Experimental setup for hyperspectral imaging and temperature stress treatments in the greenhouse. (**a**) A hyperspectral imaging (HSI) system mounted on a movable platform with a gimbal, camera stand, and Teflon reference panel for reflectance calibration. (**b**) A greenhouse heating system, featuring hot air pipes, nozzles, and fans, is used to apply controlled temperature stress. (**c**) Schematic layout of the greenhouse illustrating thermal insulation and the creation of four temperature zones (control, +1 °C, +2 °C, and +3 °C) for heat stress treatments.

To impose heat stress, a warm-air delivery system is used along the crop bench on the greenhouse’s right side. A central duct carries heated air and is fitted with evenly spaced nozzles, each calibrated to release a precise flow of hot air. A high-capacity circulation fan at the back end (the +3 °C zone) continuously draws air forward, creating a gentle airflow from the hottest section back toward the control zone. As a result, the first section (Control) remains at ambient greenhouse temperature, while successive zones are maintained at approximately +1 °C (Tr 1), +2 °C (Tr 2), and +3 °C (Tr 3) above the outdoor reference. Calibrated thermistors positioned in each zone continuously monitor air temperature throughout the experiment, providing real-time feedback to modulate nozzle flow rates and maintain the target offsets throughout the plant’s growth, from emergence through senescence. Additionally, Figure 8 presents a schematic representation of (a) the data acquisition platform used for imaging plants, (b) the greenhouse heating system heating duct and nozzles designed for heat stress treatments, and (c) the specific layout of the greenhouse used to impose heat stress on the plants.

Sweet potato plants were subjected to simulated seasonal drought and excessive water stress by carefully regulating irrigation and daily monitoring of soil moisture levels using TEROS 12 sensors (METER Group, Pullman, WA, USA). Water was supplied via drip irrigation. The stress treatments originally were divided into three categories: control, moderate, and severe stress. Control plants were maintained at a consistent soil moisture level of 30 ± 5% for the drought and excessive water stress experiments, referred to as control drought stress (CDS) and control excessive water stress (CEWS) plants, respectively. In the drought stress treatment, moderate stress (MDs) was induced by maintaining soil moisture at 20 ± 5%, while severe drought stress (SDs) conditions were defined by moisture levels falling below 15 ± 3%. Conversely, in the excessive water stress treatment, excessive moderate water stress (EMWs) was characterized by soil moisture levels of 40 ± 5%, and excessive severe water stress (ESWs) was identified when soil moisture exceeded 50 ± 5%, often resulting in symptoms of waterlogging. These carefully controlled conditions allowed for a consistent and reproducible assessment of plant responses under various water regimes.

### 4.2. Data Acquisition

Data collection in greenhouses poses unique challenges due to limited space, changing plant architecture, and the demand for flexible, high-precision imaging. To address these issues, we developed a custom mobile hyperspectral imaging platform designed explicitly for potato and sweet potato crops. The system fits easily within greenhouse rows and adapts to plant size at any growth stage, enabling rapid, non-destructive phenotyping from emergence to harvest. At its core, the platform integrates the lightweight (990 g) Senop HSC-2 Hyperspectral Camera, a rotatable, frame-based sensor that captures high-resolution images across 194 VNIR bands (511–900 nm, 1-megapixel resolution, FWHM 11 nm) at a rate of up to 74 frames per second with 12-bit digitization. Its compact footprint (199.5 × 130.9 × 97.2 mm) and low power requirements (7–17 VDC, 16 W) make it ideal for confined environments. The camera was mounted on a height-adjustable beam and stabilized with a Gremsy T3 3-axis gimbal to ensure precise orientation and consistent image quality. Hyperspectral images were acquired using the Senop HSC-2.1-C, which employed the Senop HSC-2 control software (SW version 2020.04.15.1-Retail, Senop, Oulu, Finland) to configure camera parameters (bit depth, frame rate, and storage sessions) and synchronize image capture. The imaging system was manually moved along the greenhouse rows, and each plant was individually captured weekly under natural light conditions, allowing the collection of spectral data that reflected both physiological and biochemical changes under each stress treatment. Subsequent pre-processing, visualization, and model development were conducted in MATLAB R2024a (The MathWorks Inc., Natick, MA, USA) using custom scripts.

### 4.3. Image Processing

Following image acquisition, hyperspectral images were calibrated to reflectance to compensate for variations in illumination and sensor-induced noise. Radiometric correction was performed using concurrently acquired white and dark reference images, with a Teflon tile (~99% reflectance) serving as the white reference captured under identical illumination conditions. In comparison, the dark reference was obtained by covering the camera lens with an opaque cap (~0% reflectance). The reflectance image $X_{cal}$ was computed using the following Equation (1).

(1) $$X_{cal}=\frac{X_{raw}-X_{dark}}{X_{white}-X_{dark}}$$

The $X_{raw}$ is the raw hyperspectral image, $X_{white}$ is the white reference, and $X_{dark}$ is the dark current image.

**Figure 9:**
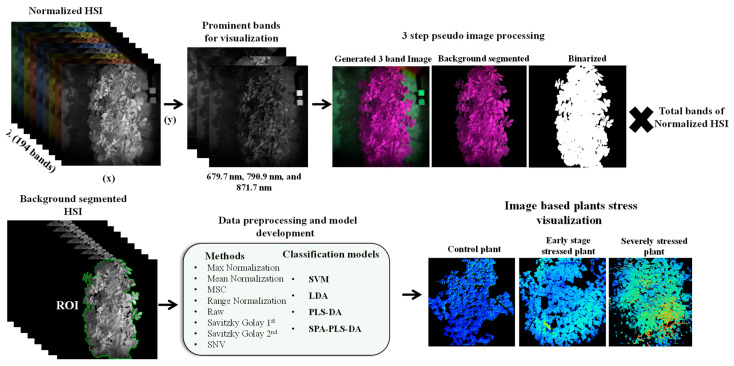
Workflow for hyperspectral image analysis. Steps include hyperspectral image acquisition, radiometric calibration, background segmentation to isolate the region of interest (ROI), spectral preprocessing, and application of classification models. Final outputs include image-based visualization maps that show the spatial patterns of stress severity.

Additionally, a pseudo-color image was constructed using three spectrally and visually discriminative bands: Band 140 (679.7 nm), Band 85 (790.9 nm), and Band 180 (871.7 nm) to facilitate the isolation of the plant region from the background. These bands were selected from the complete set of 194 based on their pronounced contrast between plant tissue and non-plant areas. The resulting composite image was enhanced and converted into the HSV color space to support robust segmentation. Empirically optimized threshold values were applied to each HSV channel to generate an initial binary mask delineating the plant region. The thresholds used were 0.223 to 0.412 for the Hue channel, 0.762 to 1.000 for the Saturation channel, and 0.001 to 1.000 for the Value channel. After thresholding, the segmented HSV images were binarized to create a refined binary mask. The binary mask was applied to all 194 spectral bands to ensure consistency across the entire hyperspectral dataset, yielding a background-free hyperspectral image cube. The resulting dataset provided a clean foundation for further analyses, including ROI spectral extraction, vegetation index calculation, and image-based visualization for stress detection via developed models. Figure 9 illustrates the background segmentation and subsequent data analysis workflow.

### 4.4. Spectral Extraction and Preprocessing

Background-free, calibrated hyperspectral images of potato and sweet potato plants were used to systematically extract spectral data from defined regions of interest (ROIs) across the VIS-NIR range, with three replicates per sample. For each cultivar, more than 3000 spectra were collected, ensuring comprehensive coverage of diverse stress conditions and physiological traits. The spectral dataset was split into 60% for calibration, 20% for validation, and 20% for the test set, which had not been used in the model training to support robust model training and performance assessment. Table 2 summarizes the distribution of extracted spectra for each experimental treatment group across calibration, validation, and test sets, as shown in the second column.

Hyperspectral data can be significantly affected by various noise sources, including light scattering, tissue structure variations, and sensor drift. Therefore, advanced preprocessing was essential to enhance spectral quality and accurately capture biochemical signatures [40]. Included techniques such as normalization, multiplicative scatter correction (MSC), standard normal variate (SNV) transformation, and Savitzky–Golay (SG) derivatives. SG was applied to reduce high-frequency noise, while MSC and SNV effectively corrected for light scattering and path length variability, leading to improved signal interpretation and model accuracy [41]. Before model development, Principal Component Analysis (PCA) was employed to visualize the spectral distribution and class structure of the extracted datasets, which comprised all control and treatment groups under both drought and temperature stress conditions.

Additionally, several widely used spectral reflectance indices were computed from the hyperspectral data to quantify the physiological responses of potato and sweet potato plants under abiotic stress conditions. These indices included the Normalized Difference Vegetation Index (NDVI), Renormalized Difference Vegetation Index (RDVI), Green NDVI (GNDVI), and Modified Chlorophyll Absorption in Reflectance Index (MCARI). Each index was calculated using specific narrowband reflectance values, primarily within the 520–900 nm range, which are sensitive to plant pigments, water content, and structural changes. The index formulas and corresponding references are listed in Equations (2)–(5) [42]. These indices subsequently evaluate and compare the stress-induced physiological changes across different treatments. The spectral bands used for index calculation were selected from the Senop HSC-2 wavelength set (510–900 nm). Specifically, the red band was taken at 670 nm (669.6 or 671.7 nm), the green band at 550 nm (550.4 or 552.4 nm), the 700 nm band at 699.9–702 nm, and the NIR band at 800 nm (799 or 801 nm). These bands were used to compute NDVI, RDVI, GNDVI, and MCARI, as given in Equations (2)–(5).

(2) $$\mathrm{NDVI}=\frac{\mathrm{NIR}-\mathrm{Red}}{\mathrm{NIR}+\mathrm{Red}}$$

(3) $$\mathrm{RDVI}=\frac{\mathrm{NIR}-\mathrm{Red}}{\sqrt{\mathrm{NIR}+\mathrm{Red}}}$$

(4) $$\mathrm{GNDVI}=\frac{\mathrm{NIR}-\mathrm{Green}}{\mathrm{NIR}+\mathrm{Green}}$$

(5) $$\mathrm{MCARI}=\left(\rho_{700}-\rho_{670}\right)-0.2\times \left(\rho_{700}-\rho_{550}\right)\cdot \left(\frac{\rho_{700}}{\rho_{670}}\right)$$

### 4.5. Model Development for Plant Stress Detection

In this study, multiple classification models were developed based on hyperspectral reflectance data to accurately identify and categorize stress conditions in potato and sweet potato plants. By leveraging PLS-DA, LDA, and SVM algorithms, the models effectively distinguish between varying levels of abiotic stress across different growth stages.

The classification decision in LDA is made by computing a discriminant function $\delta_{i}\left(x\right)$ for each class i in the specific treatment, the sample will be assigned to the class with the highest score. LDA was initially introduced by Fisher [43]. The discriminant function is given by:

(6) $$\delta_{i}\left(x\right)=x^{T}\Sigma^{-1}\mu_{i}-\frac{1}{2}\mu_{i}^{T}\Sigma^{-1}\mu_{i}+\log\left(\pi_{i}\right)$$

where the $\delta_{i}\left(x\right)$ is the discriminant function for class i, the x is the input feature vector (e.g., reflectance spectrum), while the $\mu_{i}$ is the mean vector of the class i, the Σ is the shared covariance matrix (assumed equal across classes), $\pi_{i}$ is the prior probability of class i. For LDA, the shrinkage (regularization) parameter was optimized via stratified 5-fold cross-validation on the calibration set, selecting the value that maximized mean validation accuracy.

Support Vector Machines (SVM), a robust supervised learning algorithm [44], were also employed due to their capability to handle non-linear relationships and high-dimensional spectral features. SVM identifies the optimal decision boundary by maximizing the margin between distinct stress categories, while kernel functions enable classification in transformed feature spaces [45,46]. This adaptability makes SVM highly suitable for classifying subtle spectral differences under varying environmental stressors [39,47]. In this approach, each sample’s spectral feature vector $x_{i}\in R^{p}$ was mapped into a higher-dimensional space using a kernel function $K\left(x_{i},x_{j}\right)$, enabling the separation of non-linearly separable classes. The SVM constructs an optimal hyperplane that maximizes the margin between different stress class boundaries (control, early-stage, and severe stress). The decision function in SVM is defined in Equation (7).

Since SVM inherently supports binary classification, a one-vs-rest multiclass strategy was adopted, where separate binary classifiers were trained to distinguish each class from the others. The decision function for each binary classifier is given by:

(7) $$f\left(x\right)=\mathrm{sign}\left(\sum_{i=1}^{n}\alpha_{i}y_{i}K\left(x_{i},x\right)+b\right)$$

where $\alpha_{i}$ are the Lagrange multipliers, $y_{i}\in \{-1,+1\}\;$ are the class labels, $x_{i}\;$ are the support vectors, K is the kernel function, and b is the bias term. The final class assignment was determined by selecting the class whose classifier yielded the highest decision value. The radial basis function (RBF) kernel was used, with hyperparameters optimized through stratified 5-fold cross-validation.

Furthermore, a PLS-DA model was developed to classify the obtained spectral data from potato and sweet potato plants subjected to various abiotic stress conditions. PLS-DA, an extension of Partial Least Squares Regression (PLS-R), introduced by Wold [48], is tailored for categorical response variables and enables the extraction of latent spectral features that best separate predefined stress categories. The relationship between the spectral measurements X and the class labels Y is expressed as:

(8) $$X=TP^{T\;}+Ex$$

(9) $$Y=UQ^{T\;}+Ey$$

In this study, X represents the spectral data matrix of dimension n × p, where n is the number of plant samples (potato or sweet potato) and p is the number of spectral variables. The vector b denotes the regression coefficients, and E captures the residual error terms. Preprocessed hyperspectral reflectance data were assigned to the independent matrix X. In contrast, the dependent matrix Y was encoded with categorical stress treatments. A classification threshold of ±0.5 was applied to the predicted Y values to enhance class discrimination during PLS-DA modeling, thereby facilitating a clear separation among stress categories.

The spectral data matrix and corresponding stress class labels were decomposed into latent variables (LVs) using the following forms: Where T and U are score matrices, $P^{T}$ and $Q^{T}$ are loading matrices, and E_x_ and E_y_ denote the residual errors for the spectral data *X* and matrix Y, respectively. All model hyperparameters were optimized via 5-fold cross-validation on the calibration set to prevent overfitting. In the PLS-DA models, the number of latent variables was varied up to 15, and the model with 10 latent variables was selected because it yielded the highest performance.

The performance of the classification models, including PLS-DA, LDA, and SVM, was assessed using multiple metrics: calibration accuracy (Cal-acc), validation accuracy (Val-acc), test accuracy (Test-acc), precision, recall, F1-score, and the area under the curve (AUC). Total accuracy was computed as the percentage of correctly classified samples across all classes, defined as:

(10) $$\mathrm{Precision}=\frac{TP}{TP+FP}$$

The True Positives (TP) correctly predicted the growth stage, and the False Positives (FP) incorrectly predicted the growth stage. It is expressed as follows:

(11) $$\mathrm{Recall}=\frac{TP}{TP+FN}$$

(12) $$F1=2\times \frac{\mathrm{Precision}\times \mathrm{Recall}}{\mathrm{Precision}+\mathrm{Recall}}$$

(13) $$\mathrm{Accuracy}=\frac{TP+TN}{TP+TN+FP+FN}$$

Additionally, the area under the ROC curve (AUC) was computed to evaluate the models’ discriminatory ability across various classification thresholds, with higher AUC values indicating better overall performance.

### 4.6. Effective Wavelength Selection Using SPA for Stress Classification

Hyperspectral data collected from potato and sweet potato plants within the VIS-NIR (500 to 900 nm) contain high-dimensional information, often with considerable redundancy. To address this, effective wavelength selection is crucial for simplifying the dataset, reducing computational load, and improving classification accuracy. SPA was employed to identify the most informative wavelengths associated with different stress levels in potato and sweet potato crops. SPA is a forward variable selection method that minimizes multicollinearity and redundancy by iteratively projecting variables to maximize their uniqueness [49]. This process enhances the robustness of the classification models (LDA, SVM, and PLS-DA) while retaining essential spectral features for stress discrimination.

Among the high-performing models based on the SPA, a minimum of selected bands was utilized to generate chemical images, which visualized and distinguished patterns of plant stress. Hyperspectral data were first transformed into a 2D spectral matrix, and the SPA-selected bands were multiplied by beta coefficients obtained from the resulting model. The resulting matrix was reshaped into a 3D chemical image, highlighting spatial differences across stress conditions. This process enables the pixel-wise computation of the chemical image by linearly combining spectral information weighted by model coefficients, thereby revealing the spatial distribution patterns of the target chemical or stress-related attributes. In this study, image processing, visualization, and model development were performed using a custom program developed in MATLAB (R2024a, The MathWorks Inc., Natick, MA, USA).

## 5. Conclusions

This study demonstrates the successful development and application of a greenhouse-based hyperspectral imaging system, combined with advanced machine learning, for the non-destructive detection and classification of abiotic stress in potato and sweet potato plants. By systematically simulating drought, temperature, and excessive water conditions to reflect seasonal environmental variability in South Korea, the approach enabled a precise assessment of plant physiological responses throughout the growth cycle. Severity levels were objectively defined using photoreflectance indices and PCA, which guided robust data partitioning and improved model reliability. The Integration of SPA for variable selection markedly reduces data dimensionality by as much as 85–90%, identifying as few as 21–32 key wavelengths while preserving high classification accuracy. Among the evaluated models, PLS-DA consistently outperformed SVM and LDA, achieving accuracies ranging from 90% to 98% across all stress types and plant stages, with particular excellence in differentiating between control, early, and severe stress categories. The SPA-PLS-DA models also reduced computational complexity, supporting their suitability for real-world deployment. Moreover, the generation of chemical visualization maps based on SPA-PLS-DA enabled detailed spatial interpretation of stress responses, facilitating early detection of physiological changes at the plant level. This capability is critical for non-destructive precision agriculture, empowering timely interventions, optimized resource allocation, and mitigation of yield losses under adverse conditions. Collectively, these results highlight the robustness and scalability of integrating hyperspectral imaging with advanced machine learning and variable selection techniques for early, non-invasive stress monitoring in crops. The findings underscore the potential of this approach to support sustainable agricultural management and enhance crop resilience in the face of climate variability. Nevertheless, field deployment may face challenges such as variable light conditions and interacting stress factors; therefore, future research should focus on rigorous field validation, sensor fusion, and the development of cost-effective multispectral platforms to facilitate broader adoption.

## Figures and Tables

**Table 1:** Data partitioning and number of SPA-selected wavelengths used for LDA, SVM, and PLS-DA model development and evaluation across cultivars and stress treatments.

Methods	Total Spectral	Selected Bands
DS Jopung bands 23	Calibration (2070) Validation (690) Test set (690)	564.6, 594.9, 699.9, 702, 716.1, 728.2, 740.4, 748.4, 758.5, 766.6, 770.7, 782.8, 821.2, 831.3, 833.3, 837.4, 839.4, 853.5, 859.6, 863.6, 873.7, 879.8, 881.8
DS Superior bands 24	Calibration (1865) Validation (621) Test set (622)	564.6, 594.9, 699.9, 702, 732.3, 742.4, 750.5, 754.5, 758.5, 782.8, 796.9, 809.1, 819.2, 823.2, 825.2, 829.3, 833.3, 841.4, 843.4, 855.5, 859.6, 873.7, 879.8, 885.9
DS Jopung + Superior bands 27	Calibration (3935) Validation (1312) Test set (1311)	564.6, 594.9, 699.9, 702, 714.1, 734.3, 742.4, 746.4, 750.5, 754.5, 758.5, 762.6, 766.6, 778.8, 782.8, 786.8, 796.9, 809.1, 821.2, 823.2, 829.3, 833.3, 839.4, 853.5, 855.5, 873.7, 896
TS Jopung bands 31	Calibration (2848) Validation (949) Test set (949)	556.5, 564.6, 568.6, 617.1, 627.2, 659.5, 671.7, 693.9, 708, 724.2, 728.2, 746.4, 764.6, 766.6, 799, 801, 803, 815.1, 821.2, 825.2, 833.3, 843.4, 859.6, 869.7, 873.7, 875.8, 879.8, 881.8, 883.8, 887.9, 896
TS Superior bands 32	Calibration (2385) Validation (795) Test set (795)	516.1, 556.5, 564.6, 576.7, 617.1, 619.1, 627.2, 649.4, 659.5, 671.7, 675.7, 693.9, 710.1, 744.4, 748.4, 760.6, 772.7, 778.8, 780.8, 809.1, 821.2, 823.2, 843.4, 845.4, 847.5, 857.6, 859.6, 885.9, 887.9, 893.9, 896, 898
TS Jopung + Superior bands 25	Calibration (5228) Validation (1743) Test set (1743)	556.5, 564.6, 576.7, 617.1, 619.1, 627.2, 659.5, 671.7, 675.7, 693.9, 708, 710.1, 742.4, 768.7, 817.2, 821.2, 827.3, 829.3, 833.3, 843.4, 845.4, 853.5, 855.5, 883.8, 891.9
DS Jinyulmi bands 32	Calibration (3870) Validation (1290) Test set (1290)	603, 613.1, 631.2, 671.7, 724.2, 770.7, 776.7, 778.8, 780.8, 782.8, 784.8, 786.8, 788.9, 790.9, 796.9, 803, 805, 807, 809.1, 819.2, 823.2, 847.5, 851.5, 853.5, 857.6, 861.6, 863.6, 865.6, 871.7, 881.8, 883.8, 893.9
EWS Jinyulmi bands 21	Calibration (3576) Validation (1192) Test set (1192)	625.2, 637.3, 655.5, 665.6, 728.2, 736.3, 742.4, 746.4, 752.5, 764.6, 768.7, 782.8, 794.9, 796.9, 813.1, 815.1, 823.2, 863.6, 871.7, 875.8, 877.8

Terms used in the table include DS (drought stress) and TS (temperature stress).

**Table 2:** Performance metrics of LDA, SVM, and PLS-DA models for drought and temperature stress classification in potato cultivars, including accuracy, precision, recall, F1-score, and AUC.

Cultivars	Models	Pre-Processing	Cal-acc	Val-acc	Test-acc	Precision	Recall	F1 Score	AUC
Jopung DS	LDA	MSC	96.45	96.49	96.74	0.968	0.967	0.966	0.995
	SVM	MSC	96.72	95.68	97.57	0.979	0.976	0.977	0.997
	PLS-DA	SG 2nd	99.27	98.84	99.42	0.986	0.988	0.987	0.999
SPA Jopung DS	LDA	MSC	97.73	97.67	97.34	0.977	0.977	0.977	0.990
	SVM	Smoothing	94.03	93.00	93.96	0.941	0.940	0.939	0.992
	PLS-DA	SG 2nd	98.22	97.98	98.22	0.982	0.982	0.982	0.999
Superior DS	LDA	Max-N	96.68	95.68	95.28	0.955	0.953	0.953	0.996
	SVM	Smoothing	96.39	95.63	94.98	0.952	0.950	0.951	0.993
	PLS-DA	Raw	98.34	97.34	96.71	0.970	0.967	0.968	0.998
SPA Superior DS	LDA	Mean-N	91.1	89.43	91.58	0.927	0.916	0.918	0.986
	SVM	Raw	92.51	91.65	89.79	0.904	0.898	0.899	0.978
	PLS-DA	Raw	94.44	93.54	94.14	0.943	0.941	0.941	0.989
Jopung + Superior DS	PLS-DA	Range-N	95.68	93.20	94.18	0.949	0.942	0.944	0.993
SPA Jopung + Superior DS	PLS-DA	Range-N	93.26	90.46	91.04	0.916	0.910	0.912	0.981
Jopung TS	LDA	Max-N	97.92	96.55	97.32	0.978	0.973	0.975	0.994
	SVM	Range-N	96.69	96.59	95.95	0.968	0.960	0.963	0.997
	PLS-DA	Raw	98.01	98.94	98.10	0.986	0.981	0.983	0.998
SPA Jopung TS	LDA	Max-N	91.25	90.94	90.64	0.926	0.906	0.911	0.987
	SVM	Range-N	90.54	90.49	89.44	0.924	0.894	0.902	0.988
	PLS-DA	Raw	96.73	95.38	96.26	0.973	0.963	0.966	0.996
Superior TS	LDA	SG 2nd	96.24	95.80	95.12	0.954	0.951	0.950	0.989
	SVM	SG 2nd	96.43	95.60	96.36	0.965	0.964	0.963	0.997
	PLS-DA	Range-N	99.40	99.50	99.34	0.994	0.993	0.994	0.999
SPA Superior TS	LDA	Range-N	94.39	94.59	92.52	0.933	0.925	0.926	0.991
	SVM	Range-N	94.79	93.41	94.55	0.951	0.946	0.947	0.993
	PLS-DA	MSC	97.19	96.54	96.40	0.965	0.964	0.963	0.997
Jopung + Superior TS	PLS-DA	Raw	94.72	93.56	93.99	0.944	0.940	0.939	0.998
SPA Jopung + Superior TS	PLS-DA	Raw	91.34	91.62	91.50	0.924	0.913	0.916	0.986

**Table 3:** Classification performance metrics of LDA, SVM, and PLS-DA models for drought and excessive water stress in sweet potato plants.

Cultivars	Models	Pre-Processing	Cal-acc	Val-acc	Test-acc	Precision	Recall	F1 Score	AUC
Jinyulmi DS	LDA	SG 2nd	92.77	92.01	92.86	0.931	0.929	0.929	0.988
	SVM	SG 2nd	91.8	91.45	92.47	0.913	0.905	0.907	0.989
	PLS-DA	Raw	99.56	99.84	99.45	0.995	0.995	0.995	0.999
SPA Jinyulmi DS	LDA	Range-N	90.72	90.13	90.89	0.914	0.909	0.908	0.987
	SVM	Raw	91.66	92.45	90.59	0.915	0.906	0.908	0.987
	PLS-DA	MSC	95.23	93.86	95.23	0.955	0.952	0.953	0.989
Jinyulmi EW	LDA	Range-N	96.06	94.62	95.08	0.978	0.978	0.978	0.998
	SVM	SNV	96.32	96.34	95.48	0.955	0.955	0.955	0.996
	PLS-DA	Range-N	98.99	98.13	97.81	0.978	0.978	0.978	0.998
SPA Jinyulmi EW	LDA	SNV	88.08	87.22	87.18	0.875	0.872	0.872	0.975
	SVM	SNV	89	88.99	90.33	0.905	0.903	0.903	0.977
	PLS-DA	Raw	90.83	89.83	90.53	0.908	0.905	0.906	0.978

**Table 4:** Experimental design and sampling summary.

Crop	Cultivars	Stress	Treatments Levels	Sampling Regime	Duration	HSI
Potato	Jopung, Superior	Drought	6 irrigation levels (Control 100%, Tr 1 80%, Tr 2 60%, Tr 3 47%, Tr 4 33%, Tr 5 20%)	2 GH × 6 rows × HSI 30/weekly = 360	5 weeks (April–May)	1800
Potato	Jopung, Superior	Heat	Control (daily temperature), +1 °C (Tr 1), +2 °C (Tr 2), +3 °C (Tr 3)	6 GH × 1 rows × HSI 30/week = 240	4 weeks (April–May)	720
Sweet potato	Jinyulmi	Drought	Control (CDs) 30 ± 5% soil moisture; Moderate: 20 ± 5% (MDs); Severe: < 15 ± 3% (SDs)	1 GH × 3 rows × HSI 30/weekly = 90	11 weeks (June–Sept.)	990
Sweet potato	Jinyulmi	Excessive water	Control: 30 ± 5% (CEWs); Moderate: 40 ± 5% (EMWs); Severe: > 50 ± 5% (ESWs)	1 GH × 3 rows × HSI 30/weekly = 90	11 weeks (June–Sept.)	990

Terms used in the table: Tr (treatment), GH (greenhouse), HSI (hyperspectral images), control for drought stress (CDs), moderate drought stress (MDs), and severe drought stress (SDs). Control for excessive water stress (CEWS), excessive moderate water stress (EMWs), and excessive severe water stress (ESWs).

## Data Availability

The data will be made available upon request.
